# Prediction of post-insertion infections related to totally implantable subcutaneous venous access ports in tumor patients using a nomogram

**DOI:** 10.17305/bb.2024.11583

**Published:** 2025-01-06

**Authors:** Sen Wang, Heng Zong, Lei Tang, Yuandong Wei

**Affiliations:** 1 Department of Medical Oncology, Anhui No. 2 Provincial People’s Hospital, Hefei, China

**Keywords:** Totally implantable subcutaneous venous access ports, TISVAPs, nomogram, post-insertion infections, independent risk factors, predictive model

## Abstract

Totally implantable subcutaneous venous access ports (TISVAPs) are essential for long-term central venous chemotherapy, delivering medication directly into the central veins of patients. While they play a critical role in reducing patient discomfort, TISVAPs pose a notable risk of post-insertion infections—particularly concerning for oncology patients with compromised immune systems due to aggressive treatment regimens. Our research addresses this issue by developing a predictive nomogram to estimate the risk of TISVAP-associated infections. The model is based on independent risk factors identified in our study: a history of diabetes, the type of chemotherapy, peripheral blood leukocyte count (WBC), and serum albumin levels. Using retrospective clinical data from 309 oncology patients who underwent TISVAP implantation at a tertiary A-grade comprehensive hospital, we divided the dataset into training (*n* ═ 246) and validation (*n* ═ 63) subsets. Through logistic and Lasso regression analyses, we identified the independent risk factors associated with infections. The resulting interactive nomogram demonstrated strong accuracy and reliability, with C-indexes of 0.82 and 0.835 for the training and validation sets, respectively. This tool equips healthcare providers to proactively identify high-risk patients and tailor preventive strategies accordingly. Ultimately, our research aims to enhance patient outcomes and improve the quality of life for those undergoing long-term venous chemotherapy.

## Introduction

Totally implantable subcutaneous venous access ports (TISVAPs), commonly known as “ports,” are crucial for managing patients with chronic illnesses, particularly those requiring long-term central venous chemotherapy [1–3]. These devices offer a reliable method for blood sampling and medication administration, improving patient comfort and quality of life by reducing the need for repeated invasive procedures [4, 5]. The insertion process involves creating a subcutaneous pocket, securely attaching the port to a catheter, and positioning the catheter precisely within the central venous system. This ensures stability while minimizing complications such as pocket-related issues [6]. Despite potential challenges, including central vein occlusions that may necessitate collateral vein catheterization or port repositioning [7], TISVAPs offer distinct advantages over peripherally inserted central catheters (PICCs). These advantages include a more straightforward surgical implantation process, optimal placement in the subclavian vein for efficient drug administration, greater patient mobility, and reduced irritation at the insertion site. Additionally, TISVAPs pose a lower risk of thrombosis and embolism and require less frequent maintenance, making them an effective choice for long-term management. However, post-insertion infections remain a significant risk, particularly for individuals with conditions like cancer, which often compromise immune defenses [4, 8]. Such infections, a common complication after port implantation, can occur within 30 days of surgery but are more frequently observed later [9]. Over time, infectious and thrombotic complications become the primary concerns related to ports [10, 11]. Reported incidences of long-term venous access infections range from 0.6% to 27%, with variations influenced by factors, such as catheter location, type of catheter, and patient immune status [6]. Known risk factors for port infections include patient age, performance status, the intent of treatment (palliative vs adjuvant chemotherapy), and the presence of comorbidities [8–11]. However, these factors remain subject to debate, and a mature, standardized model for assessing infection risk associated with TISVAPs has yet to be established. Properly maintained implanted port systems fall on the lower end of the infection risk spectrum. When infections do occur, they typically present as pocket or tunnel cellulitis or, more commonly, as catheter-related bloodstream infections (CRBSIs) [12]. These infections are often diagnosed either through the exclusion of other causes or by using the differential time-to-positivity method, in which blood cultures drawn from the port test positive sooner than peripheral blood samples [8, 13]. Given these challenges, developing predictive tools such as nomograms to assess post-insertion infection risks associated with TISVAPs has become a priority. These tools could significantly aid clinical decision making by helping healthcare providers identify high-risk patients and implement targeted preventive measures. The focus of this paper is to present a predictive model based on a nomogram to anticipate post-insertion infections in tumor patients with TISVAPs. By reducing infectious complications, the proposed model could improve patient outcomes and enhance the quality of life for individuals undergoing long-term central venous chemotherapy.

**Figure 1.**
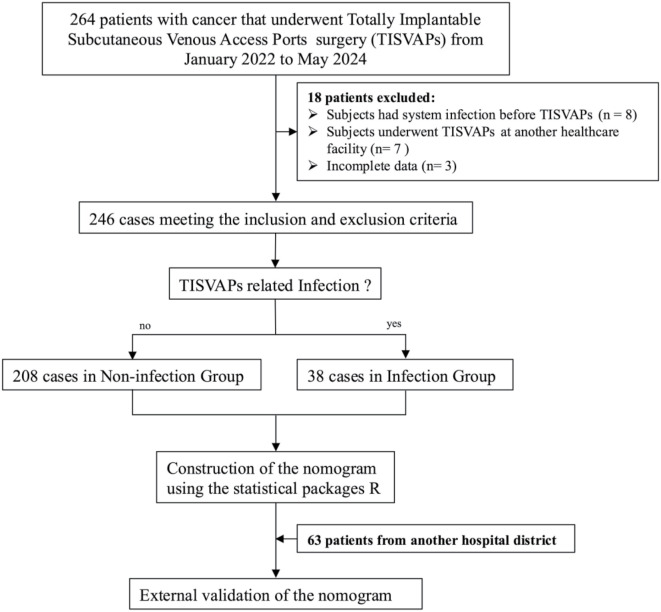
**Flowchart depicting the enrollment process for the training set and validation set in model construction.** TISVAP: Totally implantable subcutaneous venous access port.

## Materials and methods

### Patients

This retrospective study evaluated clinical cases from a tertiary A-grade comprehensive hospital across two distinct hospital districts. Data were collected from 309 oncology patients who underwent Totally Implantable Subcutaneous Venous Access Port implantation at Anhui No. 2 Provincial People’s Hospital between January 2020 and May 2024. Patients were stratified by location: those from the South District comprised the training set (*n* ═ 246), while those from the main campus constituted the validation set (*n* ═ 63). The study commenced upon patient admission for port implantation, with clinical and pathological parameters documented as baseline characteristics. The primary outcome was the presence or absence of port-related infections post-implantation. Inclusion criteria were: (1) oncology patients undergoing their first port implantation, (2) patients who used the port for intravenous chemotherapy at least once after implantation, and (3) patients with complete and up-to-date medical records. Exclusion criteria included: (1) patients whose initial port implantation occurred at another facility, (2) patients who did not receive intravenous chemotherapy through the port, (3) patients with a history of systemic or localized skin infections prior to implantation, and (4) patients with incomplete data. The data collection process is illustrated in Figure 1. Cases showing signs of catheter-related infections were reviewed, with aerobic and anaerobic cultures performed on whole blood samples. In instances of soft tissue infections, local exudate was cultured and tested for susceptibility. For severe infections deemed unsalvageable, the catheter tip was cultured on agar plates upon removal. Detailed characteristics of infected patients are provided in Table S1. Each patient signed an informed consent form for percutaneous venous catheter treatment.

### Ethical statement

This study was approved by the Ethics Committee of Anhui No. 2 Provincial People’s Hospital (Approval No. R2024-120).

### Statistical analysis

Statistical analysis and data visualization were performed using R version 4.3.0. Non-parametric tests were applied due to the non-normal distribution of the measurement data; specifically, the Mann–Whitney *U* test was used for continuous variables, and the chi-square test was used for categorical data. Lasso regression was utilized to select independent variables, while multivariate logistic regression was conducted to identify risk factors for post-insertion infections. A diagnostic nomogram model was developed using the RMS package in R. Its predictive performance was evaluated through receiver operating characteristic (ROC) curve analysis, with internal validation performed via bootstrap resampling. Calibration curves and decision curve analysis (DCA) were used to further assess and validate the model’s prediction accuracy. Statistical significance was set at *P* < 0.05.

## Results

### Analysis of baseline characteristics in the training and validation sets

This study included a total of 309 patients. By the end of the study or upon the first occurrence of a TISVAP-related infection, the overall median follow-up time was 1699 days (range: 24–1813 days). Among these patients, 50 developed infections, with nine cases classified as early infections (median: 28 days, range: 24–37 days) and 41 cases classified as delayed infections (median: 185 days, range: 44–1209 days). The average infection rate was 0.085 per 1000 catheter-days. In the training set of 246 patients, 38 cases (15.4%) of TISVAP-related infections were identified. In comparison, the validation set of 63 patients included 12 cases (19%) of TISVAP-related infections. Despite the slightly higher percentage in the validation set, statistical analysis showed no significant difference in infection occurrence rates between the two groups (*P* ═ 0.617). Table 1 provides a detailed summary of the clinical baseline characteristics, highlighting key variables for further analysis and interpretation.

**Table 1:** Baseline characteristics of patients in the training and validation sets

**Characteristic**	**Training cohort** **(*n* ═ 246)**	**Validation cohort** **(*n* ═ 63)**	***P* value**
Age (median [IQR], years)	53.50 [47.25, 59.75]	58.00 [51.50, 66.50]	0.004
BMI (median [IQR], kg/m^2^)	21.48 [19.47, 23.82]	21.72 [20.04, 23.92]	0.411
WBC (median [IQR], 10^9^/L)	5.62 [4.77, 6.88]	5.61 [3.95, 6.60]	0.047
Hb (median [IQR], g/L)	118.00 [105.00, 128.00]	111.00 [101.00, 124.00]	0.039
Platelet (median [IQR], 10^9^/L)	208.50 [149.25, 276.00]	189.00 [137.00, 241.50]	0.092
Neutrophil (median [IQR], 10^9^/L)	3.41 [2.73, 4.57]	3.38 [2.28, 4.45]	0.41
Lymphocyte (median [IQR], 10^9^/L)	1.44 [1.03, 1.82]	1.31 [0.88, 1.58]	0.026
Albumin (median [IQR], g/L)	39.90 [35.90, 42.98]	38.30 [35.45, 40.90]	0.064
PLR (median [IQR])	150.34 [99.09, 213.38]	143.18 [101.77, 218.61]	0.533
NLR (median [IQR])	2.48 [1.52, 4.07]	2.52 [1.67, 5.16]	0.385
Sex (%)			<0.001
Male	55 (22.4)	34 (54.0)	
Female	191 (77.6)	29 (46.0)	
ECOG score (%)			0.076*
0	0 (0.0)	1 (1.6)	
1	243 (98.8)	60 (95.2)	
2	3 (1.2)	2 (3.2)	
History of diabetes (%)			0.154
Non-diabetes	215 (87.4)	50 (79.4)	
Diabetes	31 (12.6)	13 (20.6)	
Dyslipidemia (%)			0.069
Non-dyslipidemia	168 (68.3)	51 (81.0)	
Dyslipidemia	78 (31.7)	12 (19.0)	
TNM_stage (%)			1
I–II	70 (28.5)	18 (28.6)	
III–IV	176 (71.5)	45 (71.4)	
Metastasis (%)			0.118
Non-metastasis	111 (45.1)	36 (57.1)	
Metastasis	135 (54.9)	27 (42.9)	
Types of chemotherapy (%)			0.402
Adjuvant chemotherapy	108 (43.9)	32 (50.8)	
Palliative chemotherapy	138 (56.1)	31 (49.2)	
Vein (%)			0.053*
Femoral	1 (0.4)	1 (1.6)	
Internal jugular	227 (92.3)	62 (98.4)	
Subclavian	18 (7.3)	0 (0.0)	
Infection (%)			0.617
Non-infection	208 (84.6)	51 (81.0)	
Infection	38 (15.4)	12 (19.0)	

The study comprises a training cohort (*n* ═ 246) and a validation cohort (*n* ═ 63), encompassing various medical parameters, including age, BMI, WBC, hemoglobin levels, platelet counts, and demographic data like sex, diabetes history, and chemotherapy types. Median values and interquartile ranges (IQR) for each characteristic are presented, along with *P* values indicating statistical significance between the two cohorts. *Fisher’s exact test. IQR: Interquartile range; NLR: Neutrophil-to-lymphocyte ratio; PLR: Platelet-to-lymphocyte ratio.

### Selection of independent risk factors through logistic and lasso regression analysis

**Figure 2.**
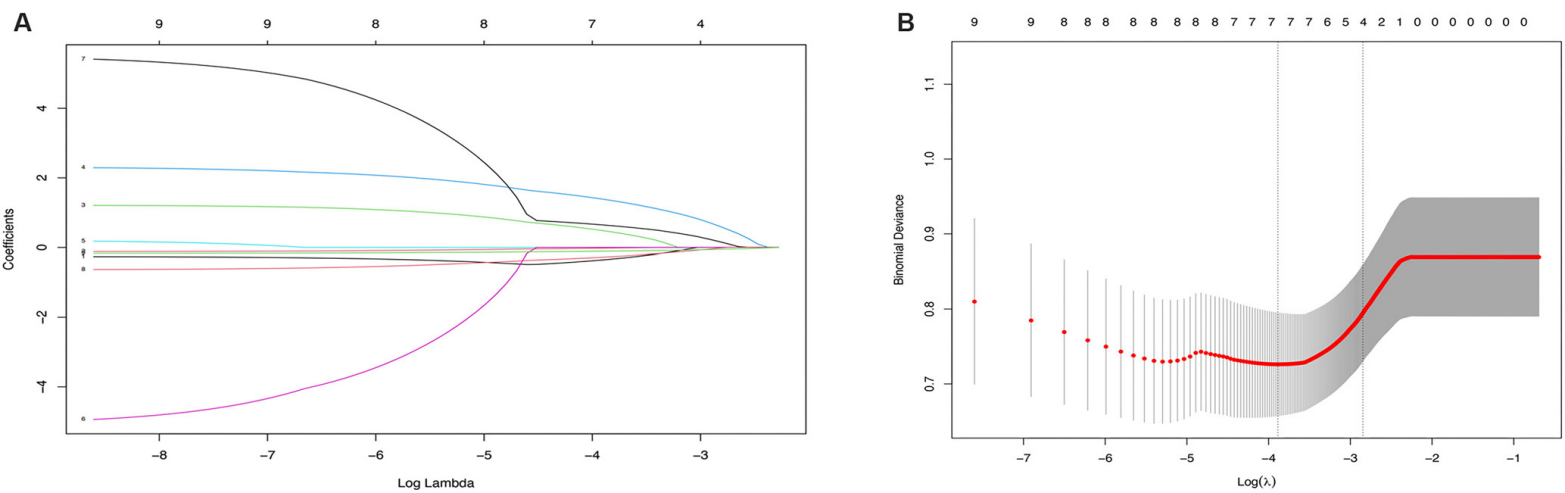
**Feature selection by LASSO regression model in training sets.** (A) The coefficients change of different genes with different lambda; (B) By verifying the optimal parameter (lambda) in the LASSO model, the partial likelihood deviance (binomial deviance) curve was plotted vs log (lambda). Dotted vertical lines were drawn based on 1 SE of the minimum criteria (the 1-SE criteria). Four features with non-zero coefficients were selected by optimal lambda.

To identify the independent prognostic factors associated with TISVAP-related infections, we utilized both logistic regression and Lasso regression analyses. The results of the univariate analysis revealed significant associations between TISVAP-related infections and several factors, including gender, BMI, ECOG performance status (ECOG_score), history of diabetes, TNM staging, metastasis, type of chemotherapy, WBC count, and serum albumin levels (Table 2). To address potential model overfitting, we applied Lasso regression, which identified four key risk factors (Figure 2). These factors—history of diabetes, type of chemotherapy, peripheral blood leukocyte count, and serum albumin level—were subsequently included in the multivariable logistic regression analysis. The final results demonstrated that all four variables were independent predictors of the risk of TISVAP-related infections (Table 3).

**Table 2:** Univariate logistic regression analysis of infection

**Variable**	**OR (95% CI)**	***P* value**
Sex	0.365 (0.176–0.773)	0.007
BMI	0.872 (0.773–0.977)	0.022
ECOG score	11.5 (1.075–251.294)	0.049
History of diabetes	4.591 (1.968–10.498)	<0.001
TNM stage	3 (1.215–9.071)	0.029
Metastasis	2.643 (1.258–5.975)	0.014
Types of chemotherapy	4.18 (1.857–10.718)	0.001
WBC	0.736 (0.552–0.938)	0.022
Albumin	0.879 (0.825–0.933)	<0.001

The table presents the odds ratio (OR) with a 95% confidence interval (CI) and *P* values for each variable, highlighting that the ECOG score, diabetes history, TNM stage, and other factors are significantly associated with infection risk.

**Table 3:** Multifactorial logistic regression analysis of post-implantation infection following TISVAPs surgery

**Variable**	**B**	**SE**	**Wald**	**OR**	**95% CI**	***P* value**
History of diabetes	1.999	0.506	3.954	7.382	2.753–20.313	<0.001
Types of chemotherapy	1.195	0.478	2.499	3.303	1.354–9.042	0.012
WBC	−0.449	0.135	−3.327	0.638	0.479–0.817	0.001
Albumin	−0.164	0.038	−4.316	0.849	0.785–0.912	<0.001

The analysis indicates that history of diabetes, types of chemotherapy, WBC, and albumin are considered important influencing factors on the likelihood of post-implantation infection following TISVAP surgery. TISVAP: Totally implantable subcutaneous venous access port.

### Establishment and clinical translation of an interactive nomogram

**Figure 3.**
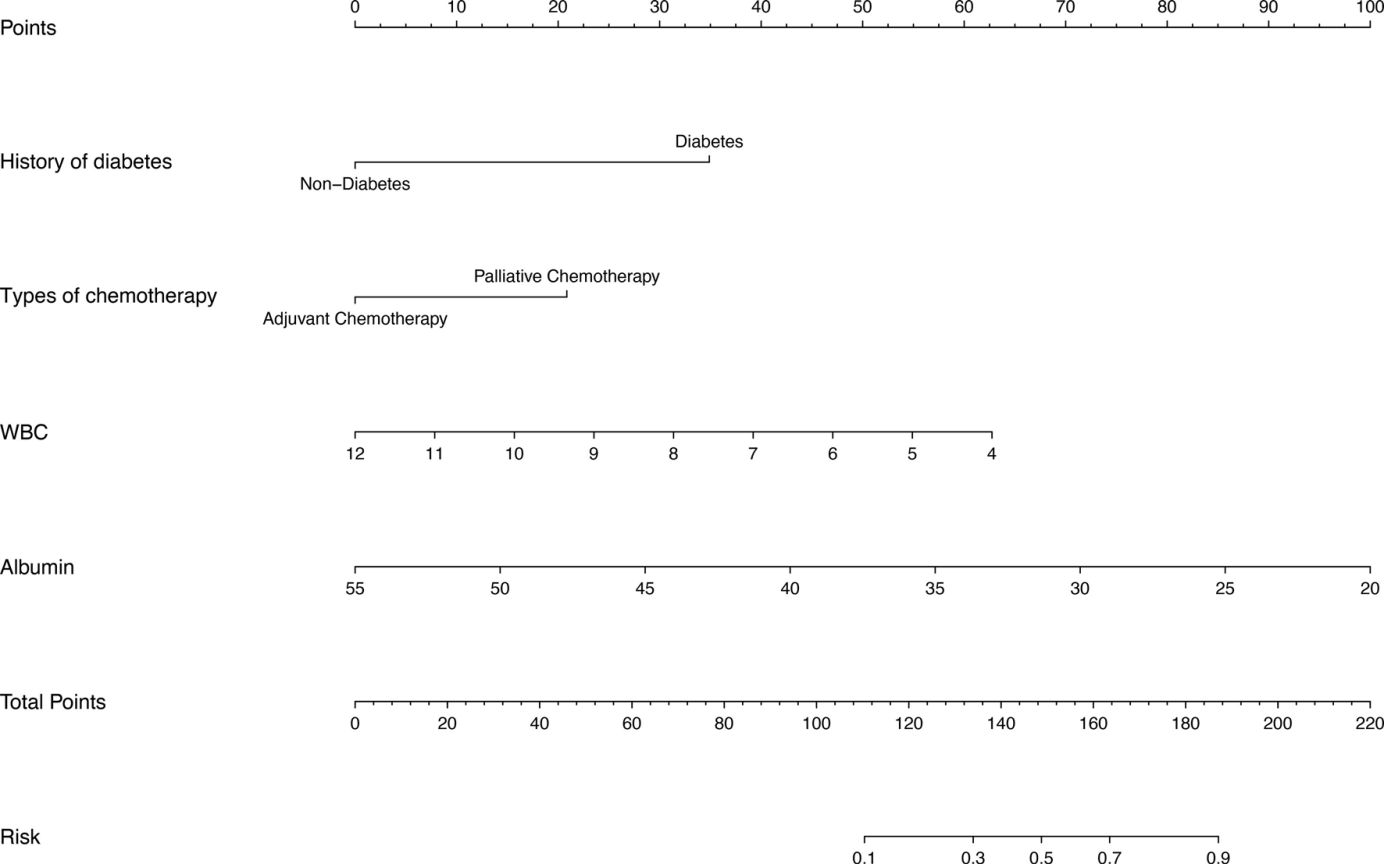
**The nomogram used to predict the probability of infection in patients with TISVAPs surgery.** TISVAP: Totally implantable subcutaneous venous access port.

The interactive nomogram developed in this study is based on four independent risk factors identified through multiple logistic regression analysis (Figure 3). The specific logistic regression model is as follows: logit (infection) ═ 4.6776 + 1.9990* History_of_Diabetes + 1.1949* Chemotherapy_types − 0.4493* WBC − 0.1637* albumin. This nomogram predicts the risk of postoperative port-related infections in cancer patients. Each risk factor (history of diabetes, type of chemotherapy, white blood cell count, and serum albumin level) is assigned a score based on the nomogram. By summing the scores, healthcare professionals can estimate an individual patient’s probability of developing a postoperative port-related infection. This tool supports risk stratification and improves clinical decision-making by enabling tailored preventive strategies for high-risk patients. Specifically, patients identified with elevated infection risk scores can benefit from proactive measures, such as enhanced monitoring for early detection of infection, strict adherence to aseptic techniques during port insertion and maintenance, and comprehensive patient education on infection prevention and proper port care. Given the lack of clear guidelines on preoperative prophylactic antibiotic use, emphasis should be placed on early detection and timely treatment of infections. For TIVAD-related infections, prompt systemic antibiotic therapy is essential, and removal of the TIVAD device may be necessary in certain cases. Overall, integrating these insights into clinical practice highlights the practical relevance of the nomogram, helping clinicians make informed decisions based on individual patient risk profiles and ultimately improving patient outcomes.

### Evaluation of the model

**Figure 4.**
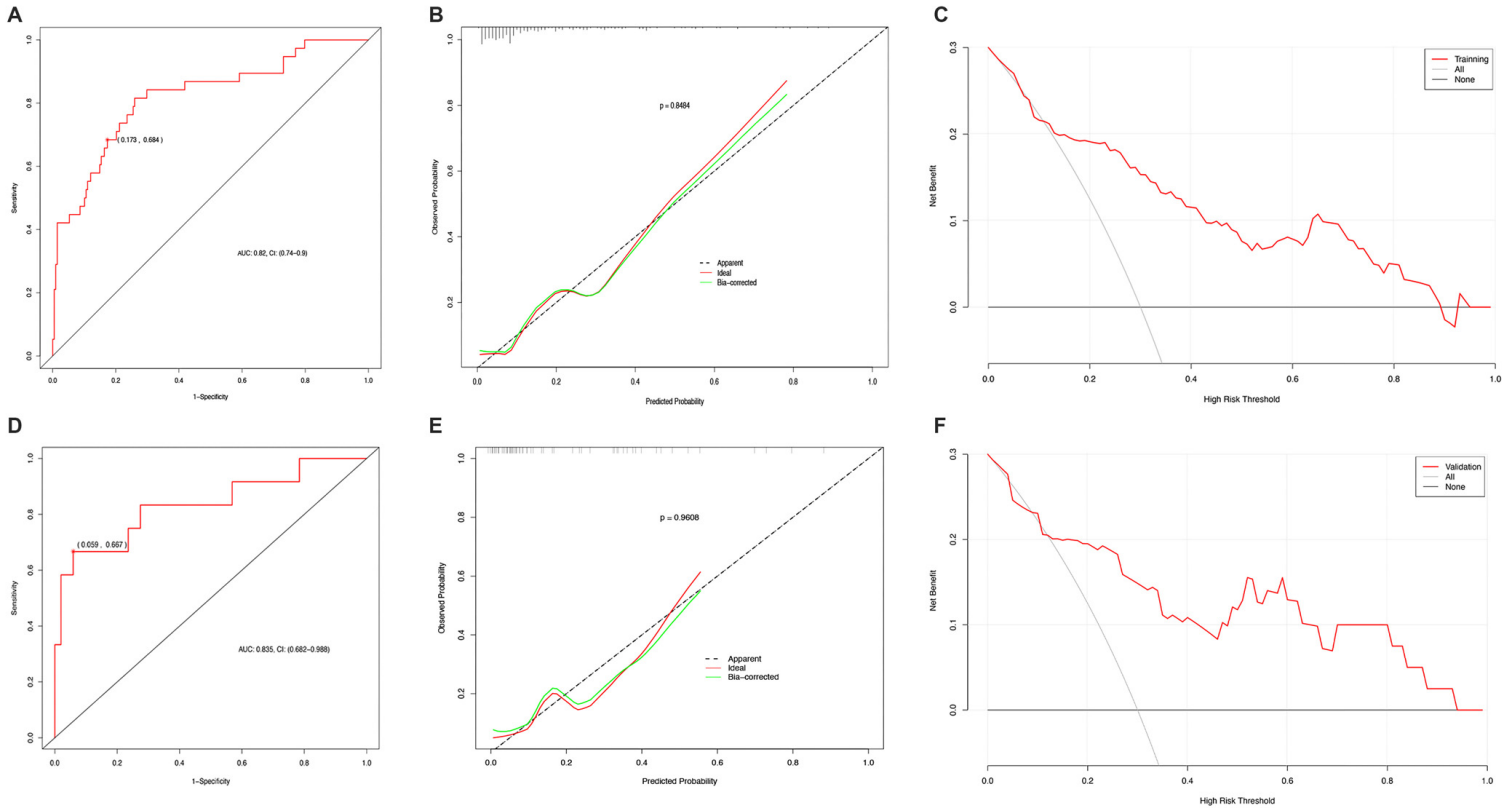
**Assessment and validation of the interactive nomogram model.** The model demonstrates high discriminatory power as shown by the AUC for both the training group, with an AUC value of 0.820 (A), and the validation group, with an AUC value of 0.835 (B). Calibration curves indicate a strong fit between predicted and observed outcomes in both the training (D) and validation cohorts (E). Decision curves further reveal the model’s clinical relevance within both cohorts, as displayed in training (C) and validation sets (F). AUC: Area under the curve.

The C-index values for the training and validation groups were 0.82 and 0.835, respectively, indicating the predictive model’s high accuracy and stability. To assess the model’s discriminative ability, ROC curves were generated for both groups, and the area under the curve (AUC) was calculated. The training group had an ROC AUC of 0.82 (95% confidence interval: 0.74–0.90), with a sensitivity of 68.4% and specificity of 82.7%. Similarly, the validation group achieved an ROC AUC of 0.835 (95% confidence interval: 0.682–0.988), with a sensitivity of 66.7% and specificity of 94.1%, further demonstrating the model’s strong predictive capacity (see Figure 4A and 4D). Calibration curves were plotted to evaluate the model’s performance, and the Hosmer–Lemeshow test was conducted to assess goodness of fit. The calibration curves for both the training and validation sets showed strong agreement between the nomogram’s predictions and observed outcomes, with corresponding *P* values of 0.8484 and 0.9608 (both *P* values > 0.05). Brier scores of 0.095 and 0.101 (close to 0) confirmed that the nomogram’s predictions for post-infusion port implantation infection probabilities aligned well with observed infection rates in the population. To further assess clinical utility, DCA was performed (see Figure 4C and 4F). Results demonstrated that, across a threshold probability range of 0.01–1, the prediction model provided a higher net benefit than the treat-all or treat-none strategies in both the training and validation sets. These findings suggest that using this nomogram to predict the risk of post-infusion port implantation infections in cancer patients can guide preventive measures, effectively reducing the likelihood of postoperative infections in clinical practice.

## Discussion

Infections related to TISVAPs are among the most common and significant complications associated with these devices, often necessitating their removal [14–16]. While early localized skin infections at the surgical wound site are prevalent, primary long-term complications typically involve bacterial or fungal colonization through three mechanisms: extraluminal migration (along the outer catheter surface), intraluminal migration (into the catheter lumen following hub contamination), and hematogenous spread (via bloodstream infections) [4, 12, 17]. A wide spectrum of microorganisms is implicated in TISVAP-related infections, with coagulase-negative staphylococci being the most common, followed by *Staphylococcus aureus* and *Candida albicans*, the leading cause of fungal infections [17–19]. Additionally, some studies have identified Gram-negative bacteria as causative agents [20]. In patients with solid tumors, port-related infections often necessitate device removal and, in severe cases, can lead to life-threatening septic shock and death [21]. Thus, prompt identification and intervention for TISVAP-related infections are essential to reduce complications, enhance survival outcomes, and improve overall quality of life. Our study’s findings align with existing research and provide further insight into specific risk factors associated with TISVAP-related infections [22–28]. These factors include a history of diabetes, type of chemotherapy administered, preoperative white blood cell count, and serum albumin levels. Understanding these risk factors is crucial for mitigating early and late infectious complications, particularly in cancer patients. The patient cohort in our study consisted predominantly of individuals with solid tumors. Although some research reports a higher incidence of infections among patients with hematologic malignancies, we hypothesize that this discrepancy may stem from the intensive chemotherapy regimens and immunosuppression characteristic of this group [21, 25, 29, 30]. Supporting this, a single-center study of 188 pediatric oncology patients identified preoperative leukocyte levels as an independent prognostic indicator of TISVAP-related infections [28]. Our findings corroborate this, revealing an inverse relationship between preoperative white blood cell count and infection risk. Serum albumin levels have also been widely associated with infection risk [31, 32]. Our study observed that lower preoperative albumin levels correlated with increased susceptibility to infection, likely due to the close relationship between albumin levels and nutritional status [33]. One major indication for port implantation in cancer patients is the need for palliative or adjuvant chemotherapy, both of which influence postoperative infection risk. Previous research found that patients undergoing palliative chemotherapy face an approximately 4.863-fold higher infection risk compared to those receiving adjuvant chemotherapy [25]. Our findings similarly indicate a 3.303-fold higher risk for patients undergoing palliative treatment. The elevated risk associated with palliative chemotherapy may be attributed to the increased frequency of treatment cycles, which significantly prolongs the duration of IVAP usage. This extended exposure elevates the likelihood of catheter-related infections, underscoring the importance of assessing infection risks in palliative care patients. While diabetes is a well-established risk factor for infections in non-oncologic populations, its impact on cancer patients remains debated. For instance, one study reported a heightened post-port implantation infection risk in cystic fibrosis patients with diabetes, while two others found no increased risk of TISVAP-related infections in diabetic cancer patients [27]. In contrast, our study demonstrated that solid tumor patients with diabetes had a 7.382-fold higher infection risk compared to non-diabetic patients, highlighting diabetes as a significant risk factor for TISVAP-related infections.

Developing predictive models is crucial for healthcare providers to assess the likelihood of TISVAP-associated infections in cancer patients, enabling timely interventions and improving patient outcomes. However, accurately predicting these infections presents significant challenges, as it requires prompt detection and thorough patient assessments to mitigate complications. Our study acknowledges several limitations that may affect the interpretation of our findings, particularly the use of a validation cohort sourced from different hospital campuses. While all participants belonged to the same institution, variations in clinical practices, patient demographics, and infection control protocols across campuses could introduce biases, thereby impacting the model’s generalizability. Furthermore, our focus on patients with solid tumors excluded those with hematological malignancies, potentially overlooking differences in infection rates between these groups. Future research should aim to include a broader spectrum of patient populations to gain a more comprehensive understanding of infection risks. Additionally, the small sample size limits the robustness of our conclusions, highlighting the importance of larger studies to enhance statistical power and validate the model in diverse populations. Future investigations should prioritize expanding the sample size and adopting multi-center study designs to capture a wider range of patient demographics and clinical environments. Such efforts would strengthen the evaluation of the predictive model’s performance and ensure its applicability across different healthcare settings, ultimately advancing strategies to prevent TISVAP-associated infections.

## Conclusion

In conclusion, the primary independent risk factors for TISVAP-related infections include a history of diabetes, the type of chemotherapy, leukocyte levels, and serum albumin concentrations. Targeting patients with multiple risk factors for early intervention is crucial to preventing these infections. The predictive model developed by our team is both straightforward and practical, offering clinicians valuable insights for proactive preoperative planning and enhancing nursing awareness of preventive measures during postoperative care. By combining preoperative and postoperative strategies and addressing high-risk factors throughout the management of TISVAPs, we can significantly improve patient outcomes and overall quality of life. This research underscores the importance of holistic, patient-centered care in TISVAP management and highlights the need for continuous evaluation and risk reduction efforts.

## Supplemental data

**Table S1:** Case characteristics of infectious patients

**Details**	**Items**	**Infection (*n* ═ 50)**
Type of infection	Soft tissue	15
	Blood stream	43
Phase of infection	*Early infection*	9
	*Delayed infection*	41
Treatment	Antimicrobial supportive therapy	47
	Removal	3
Micro-organism	Methicillin-resistant coagulase-negative Staphylococcus (MRCoNS)	11
	Methicillin-resistant Staphylococcus epidermidis (MRSE)	8
	Staphylococcus aureus	7
	Candida albicans	8
	Streptococcus pneumoniae	6
	Streptococcus species	6
	Enterococcus faecalis	1
	Klebsiella pneumoniae	1
	Non-tuberculous mycobacteria	1
	None	1

The detailed characteristics of the infected patients include the types of infections, the involved microorganisms, and the outcomes for each patient. This information provides insights into the specific infections encountered, the pathogens responsible, and the clinical results following treatment or intervention.
